# Issue des accouchements sur utérus cicatriciel dans un hôpital universitaire au Burkina

**Published:** 2012-08-02

**Authors:** Adama Dembélé, Zekiba Tarnagda, Jean Louis Ouédraogo, Oumarou Thiombiano, Moussa Bambara

**Affiliations:** 1 Centre Hospitalier Universitaire Souro Sanou. Département de Gynécologie, d'Obstétrique et de Médecine de la reproduction (DGOMR). Bobo Dioulasso, Burkina Faso; 2 Institut de Recherche en Sciences de la Santé, Bobo Dioulasso, Burkina Faso; 3 Unité de Formation et de Recherche en Sciences de la Santé, Université de Ouagadougou, Burkina Faso

**Keywords:** Utérus cicatriciels, épreuve utérine, accouchement par voie basse, check-list, cicatricial uterus, trial of labor, vaginal delivery, check-list

## Abstract

Certains auteurs ont tendance à privilégier la césarienne comme méthode de prise en charge d'une parturiente porteuse d'un utérus cicatriciel. D'autres auteurs préconisent un accouchement par voie basse quand des paramètres cliniques précis sont observés. Le but de cette étude est d'analyser la prise en charge et l'issue des accouchements sur utérus cicatriciel au Centre Hospitalier Universitaire Souro Sanou de Bobo-Dioulasso et de la comparer aux différentes approches recommandées. Nous avons menés une étude transversale dans le Département de Gynécologie d'Obstétrique et de Médecine de la Reproduction du Centre Hospitalier Universitaire Sanou Souro de Bobo Dioulasso du 1er août 2006 au 1er août 2007 et a concerné 252 parturientes ayant un utérus cicatriciel parmi 4256 accouchements déroulés pendant la même période. Les accouchements sur utérus cicatriciels ont représenté 5,92 % de l'ensemble des accouchements dans notre département. La moyenne d'âge des patientes était de 26,2 ans et la parité moyenne de 4,3. Une césarienne d'emblée a été pratiquée chez 44% des parturientes ayant un utérus cicatriciel et 56 % parmi elles ont fait l'objet d'une épreuve utérine. Sur l'ensemble des épreuves utérines, 61% des parturientes ont accouché par voie basse. La mortalité maternelle était nulle et La mortalité périnatale était relativement importante. Les conditions d'acceptabilité de la voie basse ont été les mêmes chez toutes les patientes et un check liste a été proposé pour une meilleure prise en charge. L'épreuve utérine en salle d'accouchement doit être la règle à chaque fois que cela est possible chez une parturiente porteuse d'utérus cicatriciel. L’établissement d'un check liste pour accouchement par voie basse sur utérus cicatriciel facilite les prises de décision.

## Introduction

Certains auteurs ont tendance à privilégier la césarienne comme méthode de prise en charge d'une parturiente porteuse d'un utérus cicatriciel [1]. D'autres auteurs préconisent un accouchement par voie basse si des paramètres précis sont observés [2]. Dans notre département, aucune approche de prise en charge n'est d'avance décidée concernant les parturientes porteuses d'utérus cicatriciel. Le but de la présente étude est d'analyser rétrospectivement la prise en charge et l'issue des accouchements sur utérus cicatriciel au Centre Hospitalier Universitaire Souro Sanou de Bobo-Dioulasso, de la comparer aux différentes approches recommandées et de proposer une attitude pratique pour la prise en charge des parturientes ayant un utérus cicatriciel.

## Méthodes

Une étude transversale a été conduite dans le Département de Gynécologie, d'Obstétrique et de Médecine de la Reproduction du Centre Hospitalier Universitaire Souro Sanou de Bobo du 1er Août 2007 au 1er Août 2008. D'une capacité de 100 lits et avec en moyenne 4000 accouchements par an, c'est un département à vocation universitaire, recevant les références de la ville de Bobo Dioulasso et les évacuations sanitaires venant de la région Ouest du Burkina. Ont été incluses dans l’étude toutes les patientes porteuses d'un utérus cicatriciel, reçues dans le département durant la période d’étude, pour un travail d'accouchement ou pour une césarienne prophylactique. Seule la cicatrice de la césarienne a été prise en compte excluant ainsi les cicatrices de myomectomie et les autres cicatrices opératoires sur l'utérus. Les grossesses multiples aussi ont été exclues de l’étude. La collecte des données a été faite à partir des dossiers cliniques, des registres de la salle d'accouchement et du bloc opératoire.

Dans notre département Il n'y a pas de protocole écrit sur « les accouchements et utérus cicatriciel » Il n'y a pas de monitoring fœtal au cardiotocographe, et la radiopelvimétrie n'est pas pratiquée. La surveillance du travail d'accouchement est clinique. Le check liste qui a été proposé est un ensemble de tâches que doit exécuter l'accoucheur en salle d'accouchement devant toute parturiente porteuse d'un utérus cicatriciel, en vue d'assurer une bonne surveillance du travail d'accouchement. Il a été élaboré sur la base de la revue des dossiers cliniques, de la littérature et des décisions du staff clinique le matin. L’épreuve utérine dans notre étude est définie comme une tentative d'accouchement par voie basse chez les patientes ayant subi antérieurement une césarienne. Les données collectées ont été saisies et analysées à l'aide du logiciel épi info version 6.

## Résultats

### Caractéristiques des patientes

En 12 mois nous avons colligés 252 dossiers de parturientes ayant un utérus cicatriciel sur un total de 4256 accouchements durant la même période. Nos patientes étaient des multipares, avec une gestité moyenne de 4,3 les extrêmes allant de 2 à 12 avec un intervalle de confiance de 95%. L’âge moyen des patientes était de 26,2 ans les extrêmes variant entre 15 et 42 ans avec un intervalle de confiance de 95%.

### Les cicatrices utérines

**Figure 1:**
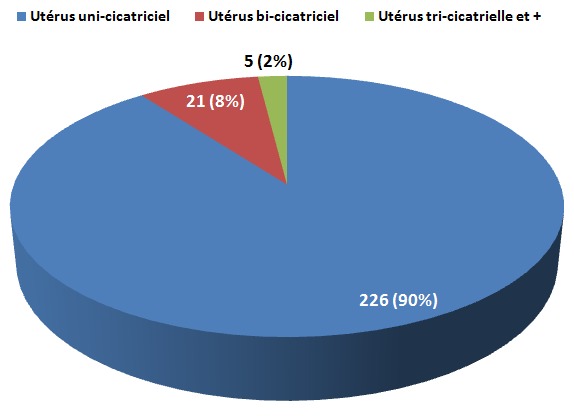
Répartition des parturientes selon le nombre de cicatrices utérines

Dans notre département 5,92% des patientes, venues pour un travail d'accouchement portaient déjà une cicatrice de césarienne. Sur l'ensemble des parturientes porteuses d'utérus cicatriciels, celles ayant un utérus uni - cicatriciel étaient au nombre de 226 soit 90% ; celles ayant un utérus bi - cicatriciel étaient 21 soit 8% de l'ensemble des utérus cicatriciels et celles ayant un utérus tri - cicatriciel et plus, étaient 5 soit 2% (Figure 1)

### Le pronostic du travail d'accouchement

La césarienne systématique : les parturientes ou gestantes qui ont subi une césarienne d'emblée, étaient au nombre de 112 soit 44% de l'ensemble des patientes ayant un utérus cicatriciel. Toutes les parturientes ayant un utérus bi ou tri - cicatriciel ont subi systématiquement une césarienne. Les indications de césarienne systématique ont été posées en salle d'accouchement ou en salle de consultation prénatale, et la césarienne a été faite ensuite (Tableau 1).

**Tableau 1:** Indications systématiques de césarienne sur utérus cicatriciel

Indications	Dystocie osseuse	Utérus Bi-cicatriciel	Pré éclampsie sévère	Gros fœtus	Siège	Utérus Tri-cicatriciel et plus	Autres	Total
Nombre	50	21	15	8	7	5	6	112
Pourcentage	45%	19%	14%	7%	6%	4%	5%	100%

**Figure 2:**
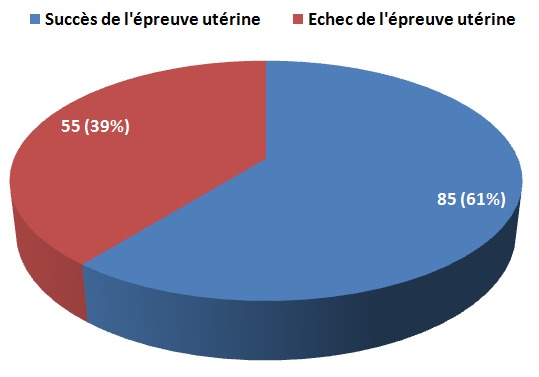
Pronostic de l’épreuve utérine

L’épreuve utérine : sur 252 parturientes ayant un utérus cicatriciel, il a été indiqué une épreuve utérine chez 140 d'entre elles, soit 56 % de l'ensemble des utérus cicatriciels. Toutes avaient un utérus uni - cicatriciel. Sur les 140 parturientes ayant subi l’épreuve utérine, 85 parturientes ont pu accoucher par voie basse sans difficulté majeure. Cela représente 61 % de succès de l’épreuve utérine, contre 39 % d’échec de l’épreuve utérine (Figure 2). Il n'y a pas eu d'induction du travail sur utérus cicatriciel dans notre département.

Les conditions théoriques d'acceptation de l'accouchement par voie basse ont été les suivantes : Grossesse unique, présentation céphalique, absence de cicatrice corporéale dans la mesure o[ugrave] le protocole opératoire est connu, poids fœtal estimé à l’échographie inférieur à 3800 grammes, bassin cliniquement normal, placenta non recouvrant, absence de souffrance fœtale ou de tout autre urgence obstétricale, présence continuelle du personnel médical en salle de travail .

Les échecs d’épreuves utérines : Il y a eu 55 cas d’échecs d’épreuves utérines, soit 39% de l'ensemble des épreuves utérines. Ces parturientes n'ont pas accouché par voie basse et ont subi une césarienne ou une laparotomie. Les Causes d’échec de l’épreuve utérine sont dominées par la dystocie dynamique et la souffrance fœtale aigue (Tableau 2). Les indications de laparotomies, étaient 5 cas de ruptures utérines soit 3% de l'ensemble des 140 cas d’épreuves utérines.

**Tableau 2:** Causes d’échec de l’épreuve utérine

Causes de l’échec de l’épreuve utérine	Dystocie dynamique	Souffrance fœtale aigüe	Défaut d'engagement	Rupture utérine	Suspicion de déhiscence	Total
**Nombre**	18	16	15	5	1	55
**Pourcentage**	33%	29%	28%	9%	1%	100%

### Le pronostic fœtal et maternel

Il n'y a pas eu de décès maternel. Il y a eu 12 morts fœtales constatées au décours du travail d'accouchement. Ces morts fœtales s'expliquent par 5 cas de ruptures utérines, 1 procidence du cordon, 4 souffrances fœtales réanimées en vain, et 2 morts fœtales dont les causes ne sont pas retrouvées.

## Discussion

L'attitude à adopter devant une parturiente porteuse d'utérus cicatriciel a évolué au cours du siècle. Il faut choisir entre les deux extrêmes qui ont tendance à rendre à 100% le taux de césarienne itérative ou au contraire à rendre le pourcentage des césariennes comparable à celui des secondipares non césarisées [1–6]. Nous avons mené une étude transversale sur une période de douze mois sur les utérus cicatriciels et accouchement par voie basse.

Dans notre département 5,92 % des patientes qui viennent pour un travail d'accouchement, sont porteuses d'une cicatrice au moins, de césarienne. Ce taux est plus bas dans des études antérieures, menées par, Cissé avec 1,5 % [7] et Tshilombo avec 2,4 % [8]. Notre proportion élevée peut s'expliquer par le fait que nous sommes une structure universitaire o[ugrave] est référée la majorité des accouchements à risque dont les accouchements sur utérus cicatriciels.

Sur l'ensemble des parturientes ayant un utérus cicatriciels, 44 % vont subir systématiquement une césarienne et près de la moitié d'elles, pour une indication permanente de dystocie osseuse. Lieberman trouve 24 % pour les césariennes systématiques [9], Mercer B trouve 32 % [10] et nous avons le même taux que Cissé [7]. La variabilité des taux en fonction des études se justifie par la variabilité des indications systématiques de césarienne sur utérus cicatriciel. Les deux indications de césarienne systématique sur utérus cicatriciel qui restent communément retrouvées dans la littérature, sont les dystocies osseuses et la présentation du siège [6–13].

Sur l'ensemble des accouchements dans notre département, 1% des parturientes vont être systématiquement césarisées et répondent ainsi à la célèbre maxime de Racinet Claude: césarienne un jour, césarienne toujours [14].

Aucune épreuve utérine n'a été tentée chez les parturientes ayant un utérus bi ou tri - cicatriciel. Cette attitude est celle qui semble retenue dans des études antérieures faites en Afrique de l'Ouest sur le même sujet [7, 11]. Bernard blanc à Marseille trouve un taux de succès de 60 % d'accouchement par voie basse dans les épreuves d'utérus bi - cicatriciels, sur une série de 115 cas, avec induction du travail dans 18 % des cas, par amniotomie et perfusion d'ocytocique [4]. Les conditions de surveillance du travail ont été particulières avec : la radio pelvimétrie systématique, la tocométrie interne, une connaissance parfaite de la qualité des cicatrices antérieures. Ces conditions font défaut dans notre contexte.

Dans notre département, 56 % des parturientes porteuses d'un utérus cicatriciel subissent une épreuve utérine. Toutes celles qui subissent l’épreuve utérine ont des utérus uni - cicatriciels et 61 % d'entre elles vont accoucher par voie basse. Philippe Boisselier après avoir comparé les études de 7 auteurs trouve une moyenne de 45,7 % de succès d’épreuve utérine [1]. Ainsi comme c'est indiqué Tableau 3, il présente les taux de succès des épreuves utérines qui varient entre 32 et 93 % avec comme dénominateur l'ensemble des épreuves utérines et comme numérateur le succès des épreuves utérines [7, 10–17]. Les attitudes aussi varient, mais les auteurs sont presque tous unanimes sur un point : il faut privilégier la voie basse, même avec les utérus bicicatriciels [3, 7, 10–17].

**Tableau 3:** Pourcentage des épreuves utérines réussies en fonction des études

Auteur	Cissé	Hibbard	Hamet	Mercer	Hassan	Landon	Kraiem	Tripathi	Notre série
Pays	Sénégal	USA	Niger	France	USA	USA	Tunisie	Inde	Burkina
Taux d’épreuve utérine réussie	85%	93%	32,4%	66%	67,2%	73,6%	76,1%	73%	61%
Année de l’étude	1999	2001	2001	2004	2005	2005	2006	2006	2007

Il n'y a pas eu de déclenchement du travail sur utérus cicatriciel. Cette attitude se justifie par la peur de la rupture utérine dans les déclenchements sur utérus cicatriciels, d'autant plus qu'il y a souvent une méconnaissance de la qualité de cette cicatrice dans notre contexte. Muteganya a utilisé l'amniotomie et les ocytociques comme moyen d'induction du travail sur utérus fragilisé chez des patientes dont la moyenne de parité était de 5 [18]. Bernard Blanc a utilisé l'amniotomie et la perfusion d'ocytocique avec succès chez des parturientes ayant des utérus bicicatriciels [4]. Nous restons prudents par rapport aux utérus bi - cicatriciels et l'attitude selon notre étude reste la césarienne systématique à toute patiente porteuse d'un utérus doublement cicatriciel. Un check liste a pu être ainsi proposé à partir de notre étude pour les accouchements sur utérus uni cicatriciels :

1) Poser le diagnostic ou non d'une urgence obstétricale et agir en conséquence ; 2) Dans les antécédents de la patiente, avoir le maximum de renseignements sur la cicatrice utérine : espace intergénésique, indication, protocole opératoire, suites opératoires ; 3) Faire un examen obstétrical soigneux en insistant sur le bassin et la présentation du fœtus : ogive pubienne, promontoire, ligne innominée, épine sciatique ; 4) Poser un diagnostic précis. (Par exemple : début de travail d'accouchement sur utérus uni cicatriciel chez une patiente de 30 ans, G2P1) ; 5) Soumettre le dossier au médecin de garde ; 6) Pendant toute l’épreuve utérine, la présence constante en salle de travail de personnel médical et l'utilisation du partogramme pour la surveillance du travail est obligatoire ; 7) La perfusion de syntocinon quand elle est permise est ordonnée par un médecin ; 8) Alerter le médecin de garde devant tout saignement ou douleur permanente de la cicatrice en cours de travail ; 9) Au moment de l'expulsion l'utilisation des instruments d'extraction est conseillée ; 10) Ne pas faire des expressions manuelles sur l'utérus au moment de l'expulsion fœtale ; 11) La révision utérine n'est pas systématique après l'accouchement ; 12) La surveillance du post-partum immédiat durant les deux heures juste après l'accouchement doit être rigoureusement menée.

## Conclusion

Plus de la moitié (56%) des patientes qui viennent dans notre département pour y accoucher et qui ont un utérus uni cicatriciel ont été autorisées à accoucher par voie basse. Il y a eu 61 % de succès à ces épreuves utérine dans un contexte d'absence de protocole et de check liste. On pourrait avoir des résultats meilleurs si l'on tient compte des recommandations de la présente étude. L'induction sur utérus cicatriciel est faisable et n'entraîne pas forcément plus de rupture utérine. Les conclusions de cette étude rejoignent la majorité des études qui ont déjà été faite sur le sujet: Il faut toujours privilégier la voie basse sur les utérus cicatriciels tant que cela est possible.
